# Sysmex UN2000 detection of protein/creatinine ratio and of renal tubular epithelial cells can be used for screening lupus nephritis

**DOI:** 10.1186/s12882-022-02953-x

**Published:** 2022-10-05

**Authors:** Yabin Chen, Yuan Zhao, Zhishan Zhang, Xiang Cheng, Jie Lin, Jiaming Li, Yibo Wu, Zhen zhong Lin, Jing Jing

**Affiliations:** 1 Clinical Laboratory, Quanzhou First Hospital Affiliated to Fujian Medical University, Quanzhou, China; 2 Department of Clinical Laboratory Medicine, Xijing Hospital, Fourth Military Medical University (Air Force Medical University), 127 Changle West Road, 710032 Xi’an, Shaanxi China

**Keywords:** P/C, RTEC, Systemic lupus erythematosus, Lupus nephritis, UN2000

## Abstract

**Objectives:**

This study is aimed to evaluate if automated urine sediment analysis UN2000 can be used to screen lupus nephritis.

**Methods:**

UN2000 was used to examine 160 urine samples from patients with systemic lupus erythematosus and 124 urine samples from Lupus nephritis. The result of protein/creatinine ratio(P/C) and renal tubular epithelial cells (RTEC) were evaluated. With biochemical analysis and microscopic examination as the gold standard, the Kappa consistency test was used to analyze the accuracy of P/C and RTEC. Analysis was to evaluate the accuracy of P/C single item or RTEC single item and both screening lupusnephritis.

**Results:**

The consistency of P/C and the gold standard, and that of RTEC and the gold standard are respectively strong and good (0.858 vs. 0.673). The specificity, positive predictive value, and coincidence were the highest when P/C ≥ 2 + was set as the only screening standard for lupus nephritis. When the standard was selected between P/C ≥ 2 + or RTEC > 2.8 cells/µl, the sensitivity and negative predictive value were the highest.

**Conclusion:**

UN 2000 can be used to screen lupus nephritis by detecting P/C and RTEC.

## Introduction

Lupus nephritis (LN) is a common complication following systemic lupus erythematosus (SLE) in most patients, featured in different pathological types of kidney damage. In China, half of SLE patients have LN, with an incidence of the disease higher than that among white people[1, 2]. LN is China’s most common secondary immune glomerular disease[3, 4]. Primarily within 5 years from the diagnosis, there still presents a rate of progression to end-stage renal disease (ESRD)[5]. LN can often be linked to mortality in SLE[6]. Therefore, in SLE patients, the occurrence of LN should be identified as soon as possible, and renal biopsy should be carried out; it is conducive to early intervention of SLE and also the key to ensuring a favourable prognosis of patients[7].

The diagnostic criteria of LN involve urine protein, creatinine, and urine sediment analysis, so a routine urine examination is one of the keys to early detection of LN. Automated urine sediment analysis by UN2000 is representative of the urine analyzers produced by Sysmex Corporation, Japan. UN2000 includes UC3500 urine dry chemistry analyzer and UF5000 urine sediment analyzer. Based on the original test parameters, new parameters such as protein/creatinine ratio (P/C) and renal tubular epithelial cells (RTEC) are added by Sysmex Corporation. These two parameters have been proved to be one of the indicators of renal injury, even one of the indicators of secondary acute renal injury [8–11]. Therefore, they may also be used for early detecting LN patients complicated with renal injury from SLE patients. The present study aims to detect urine samples of 160 SLE patients and 124 SLE patients with LN by UN2000 and conventional microscopic examination, respectively. Comparing the accuracy and consistency of the P/C and RTEC, the study further evaluates the two parameters’ ability to screen LN.

## Materials and methods

### Urine specimens

284 urine specimens were randomly selected among SLE patients from the department of rheumatology and immunology in Quanzhou First Hospital between January 2021 and December 2021. The patients were females whose ages ranged from 13 yrs to 73yrs with the median age being 36 years. The protocol was approved by the Ethics Committee of Quanzhou First Hospital (China), and the protocol number is [2021] 193. According to the incidence of renal injury complicated, they were divided into the SLE group (163 females, aged 13–66 years, median age 34 years) and the LN group (124 females, aged 20–73 years, median age 37 years). Meanwhile, healthy women were selected as the control group (336 females, aged 21–69 years, median age 37 years).

### Inclusion standards

SLE group: (1) Patients who were with a final diagnosis of SLE made by the clinician in accordance with the 2012 systemic lupus erythematosus international lupus research clinical collaboration group (SLICC) or SLE classification standard formulated by EULAR / ACR in 2019[12]. (2) Patients were excluded when they were with diabetes, allergic purpura and other diseases that may cause acute and chronic kidney injury, or patients with kidney disease and other urinary system diseases. (3)Proteinuria stably lower than or equal to the level of 500 mg/24 h, or random proteinuria < 3+, or P/C ≤ 500 mg/g(50 mg/mmol). (4) Microscopic examination of urine sediment showed no cell cast (erythrocyte cast, haemoglobin cast, granular cast, tubular cast, and mixed cast) or no active urine sediment (white blood cells excluding ≤ 5/HP, red blood cells ≤ 5/HP, excluding urinary tract infection).

LN group: (1) Clinically diagnosed as SLE, (2) Patients were excluded when they were with acute and chronic kidney injury caused by diabetes, allergic purpura, or with nephropathy and other urinary system diseases. (3) Proteinuria stably overcame the level of 500 mg/24 h, or random proteinuria 3+, or P/C ratio > 500 mg/g(50 mg/mmol)[7]. (4) Microscopic examination of urine sediment showed several kinds of cell cast (erythrocyte cast, haemoglobin, granular cast, tubular cast, and mixed cast), or active urine sediment (white blood cells excluding > 5/HP, red blood cells > 5/HP, excluding urinary tract infection).

Control group: (1) Normal renal function and urine test. (2) Normal imaging examination and physical examination. (3) No acute and chronic kidney injury caused by diabetes, allergic purpura, or patients with nephropathy and other urinary system diseases. (4) No history of systemic diseases such as hepatitis, rheumatism, and urinary system diseases.5) No history of major surgery, 6) Not taking drugs in recent one month, 7) Non-menstrual.

### Sample detection

Mid-stream specimens were collected in sterile containers and were tested within two hours. Each specimen was divided into two sterile tubes, with 10ml urine being tested by Beckman AU5800 automatic biochemical analyzer and the other tube of 10ml urine by UN2000. Urine protein, creatinine, P/C, and RTEC were detected using both analyzers.

### Conventional microscopic examination

8ml of urine sample was centrifuged for 5 min at 400 g (1500 rpm) before the supernatant fluid was taken out and about 100ul sediment was left. After mixing the sediment, 1 drop of S-M (sternheirnermalbin) staining solution was added for one-minute staining. After that, approximately 20µl was put onto a microscope slide which was then examined under a CX23 microscope (Olympus, Tokyo, Japan) by 2 medium-grade professionals titled clinical laboratory technicians. The average value of the two measurements for RTEC was calculated. The two technicians had completed the personnel comparison; the coincidence rate ≥ of 80% represented passing.

### Automated urine sediment analyzers and reagents

UN2000 was provided by Sysmex Corporation, Japan. AU5800 was provided by Beckman Coulter Corporation, American. Sysmex Corporation provided all reagents, quality control material, and calibrator. All of them were used within the validity period. Regular calibration and performance verification had been previously performed. The urine was detected everyday after passing the quality control whose result conformed to ISO 15189. TD4N centrifuge (low-speed urine centrifuge) was provided by Shanghai Luxiangyi centrifuge corporation. S-M staining solution was purchased from Baso Corporation, Zhuhai, China. CX23 binocular microscope was supplied by Japan Olympus Corporation, Japan.

### Statistical analysis

#### Accuracy evaluation of P/C

The P/C results detected by AU5800 were used as the gold standard. According to the UN2000 test strip instruction book, the instrument result is negative when P/C < 150 mg/g, which is positive (+) when P/C (150–499) mg/g and positive (2+) when P/C ≥ 500 mg/g. Kappa’s concordance coefficient was calculated for the agreement between the instruments. Kappa values were considered to be poor agreement (0 ~ 0.20), fair (0.21 ~ 0.40), moderate agreement (0.41 ~ 0.60), good agreement (0.61 ~ 0.80) and excellent agreement (0.81 ~ 1.00).

#### Accuracy evaluation of RTEC

Establishment of reference intervals and verification of RTEC.

RTEC results were extracted from the control group of 316 healthy women to establish the reference. The detection data were non-normally distributed. Consequently, referring to the CLSI C28-A3 document [13], (0-*P*_95_) was set as the reference range after excluding outliers in the data while 20 healthy women were selected from the control group to verify the reference interval. 90% of the test results passed the verification within the established reference range.

With the conventional microscopic results as the golden standard, positive was defined when RTEC was found by conventional microscopic examination, while it was negative when no RTEC was detected. When the results of UN2000 were greater than the reference range established in this study, the results were positive. Otherwise, the results were negative [11]. The negative and positive coincidence rates were analyzed by Kappa consistency test.

#### Comparison of RTEC levels in the control group, SLE group, LN group, and ROC curve analysis

SPSS21.0 was used for statistical analysis. The results of RTEC were expressed by mean and standard deviation. Kruskal Wallis test was used for comparison among groups. Mann Whitney U test was used for comparison between groups. The receiver operating characteristic (ROC) curve was used to analyze the ability of RTEC to screen LN. A further analysis was performed on the diagnostic efficacy of single item or combination of P/C and RTEC for screening LN.

## Result

### Accuracy evaluation of P/C tested by UN2000

With results of AU5800 as a golden standard and Kappa = 0.858, the consistency of UN2000 and AU5800 was very high as shown in Table 1. According to UN2000 test strip instructions, ‘-’ represents P/C < 150 mg/g, ‘1+’ represents P/C(150 ~ 499)mg/g, and ‘2+’ represents P/C ≥ 500 mg/g in the Table which shows that the judgment errors of UN2000 results are within ± 1, with no result being ‘-‘ or misjudged as ‘2+’ and non being ‘2+’ or misjudged as ‘-‘.

**Table 1:** Accuracy evaluation of P/C tested by UN2000.

Test parameters		UN2000*				Kappa
		**—**	**1+**	**2+**	**Total**	
AU5800(mg/g)	< 150	427	21	0	448	0.858
	150 ~ 490	4	48	8	60	
	≧ 500	0	7	105	112	
	Total	431	76	113	620	

### Reference range of RTEC

The urine RTEC results of healthy women were non-normally distributed. The reference range was 0-2.5 cells/µl. 20 healthy female urine samples were selected to verify the reference range, and the validation passed.

### Consistency of RTEC tested by UN2000 and by manualmicroscopy

Conventional microscopic examination was considered the golden standard technique with Kappa = 0.673. Table 2 shows a very high consistency of UN2000 and conventional microscopy. The test of UN2000 was seen as positive when the result was > 2.5 cells/µl and negative when ≤  2.5 cells/µl.

**Table 2:** Accuracy evaluation of RTEC tested by UN2000

Test parameters		UN2000*				Kappa
		**negative**	**positive**		**Total**	
Conventional microscopy	negative	470	48	518		0.673
	positive	15	87	102		
	Total	485	135	620		

### Comparison of RTEC results in the control group, SLE group and LN group

**Fig. 1:**
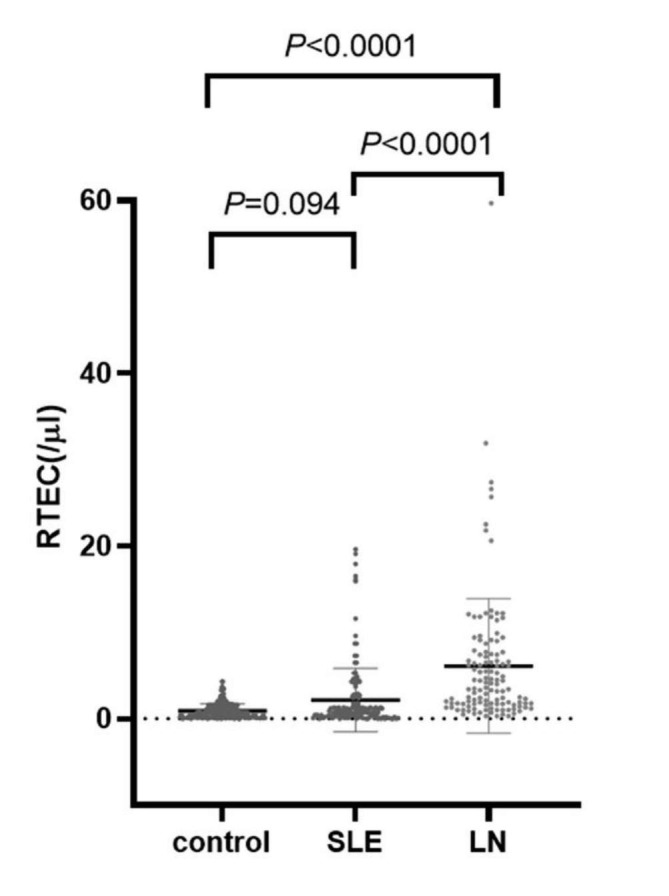
Results of RTEC in the control group, SLE group and LN group, respectively. The results were not statistically significant between the control and SLE groups (*P* = 0.094). But there was a substantial difference between the control group and the LN group (*P* < 0.0001), and there was also a difference between the SLE group and LN group (*P* < 0.0001)

Results of RTEC in the control group, SLE group, and LN group were (0.92 ± 0.85) cells/µl, (2.20 ± 3.73) cells/µl, (6.12 ± 8.88) cells/µl, respectively. Figure 1 indicates no statistical significance between the control and SLE groups (*P* = 0.094). There was a substantial difference between the control group and the LN group (*P* < 0.0001), so as between SLE group and LN group (*P* < 0.0001).

### ROC curve analysis

**Fig. 2:**
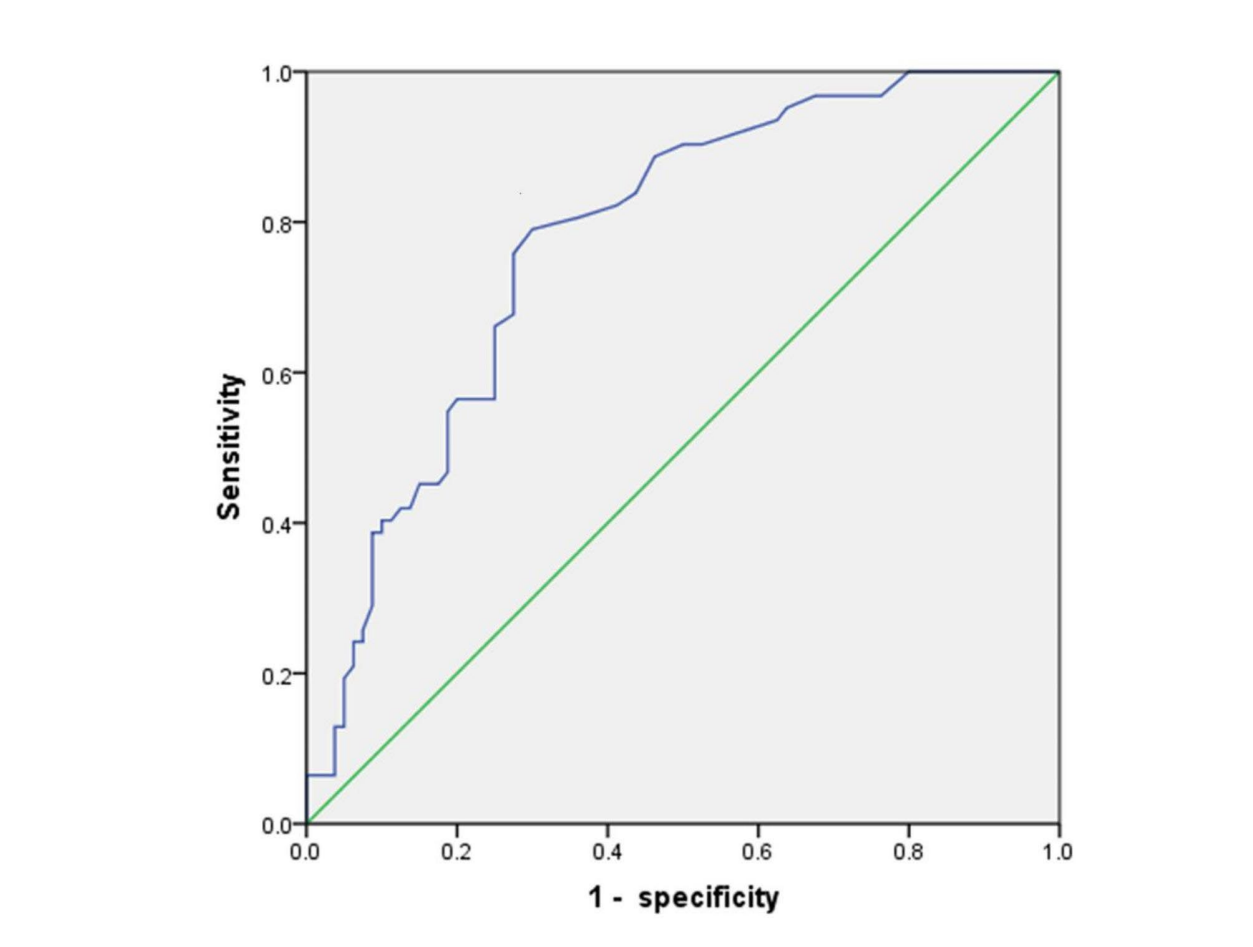
ROC curve for RTEC of LN screening. The RTEC count presents high area under curve (AUC) values in the ROC analysis (AUC = 0.777).

With the LN group as the positive group and all groups as research targets, ROC analysis was conducted to analyze the diagnostic ability of RTEC in screening LN from SLE patients. The area under the curve (AUC) for RTEC is 0.777 (95%CI = 0.702–0.853). The ROC curves are shown in Fig. 2. The Screening performance was offered at a cut-off of 1.35 cells/µl, and Youden index was 0.490. Since the result of 1.35 cells/µl was less than the reference range of 2.5 cells/µl, the study further selected some cut-off values with RTEC > 2.5 cells/µl according to the Youden index to evaluate the corresponding diagnostic efficacy. The result showed that when the cut-off value was 2.8 cells/µl, the negative predictive value (NPV) of LN screening and the coincidence were the highest.

**Table 3:** Performance evaluation of screening LN with different RTEC cut-off values

performance index	cut-off (cell/µl)							
	2.55	2.65	2.80	3.05	3.25	3.35	4.35	4.80
Youden index	0.327	0.340	0.365	0.361	0.329	0.313	0.302	0.303
sensitivity (%)	59.68	59.68	59.68	58.06	54.84	53.23	48.39	43.55
specificity (%)	76.25	77.50	80.00	81.25	81.25	81.25	85.00	90.00
PPV (%)	66.07	67.27	69.81	70.59	69.39	68.75	71.43	77.14
NPV (%)	70.93	71.26	71.91	71.43	69.89	69.15	68.00	67.29
coincidence (%)	69.01	69.72	71.13	71.13	69.72	69.01	69.01	69.72

### Comprehensive assessment of P/C and RTEC screening LN

According to the ROC curve analysis, the optimum RTEC cut-off value is 2.8 cells/µl. According to the diagnostic criteria of LN, P/C ≥ 2 + and RTEC > 2.8 cells/µl are the positive standard for evaluating the screening competence of UN2000. The result showed that the specificity, positive predictive value (PPV), and coincidence of individual P/C screening LN were the highest. The sensitivity and negative predictive value were the highest when P/C or RTEC was positive (Table 4).

**Table 4:** Performance evaluation of P/C and RTEC screening LN

screening program	sensitivity(%)		specificity(%)		PPV(%)	NPV(%)	coincidence(%)		
P/C	87.90	97.50		96.46		91.23		93.31	
RTEC	59.68	80.00		69.81		71.91		71.13	
Either RTEC or P/C positive	95.97	80.00		78.81		96.24		86.97	
Both RTEC and P/C positive	50.81	97.50		94.03		71.89		77.11	

## Conclusion

RTECs are exfoliated cells in proximal or distal urine segments derived from renal tubules, and they are with diagnostic potential as a tangible component in urine[14]. Research shows RTEC has specificity in renal tubular injury, and its detection in a urinary sample could allow early recognition of renal injury [10, 11]. Therefore, it is a good non-invasive index. However, there is insufficient attention to the detection and clinical significance of RTEC due to cumbersome and inefficient conventional microscopic examination and high technical requirements for clinical inspectors to identify the RTEC form. Consequently, it is difficult to carry out a mass microscopic examination. Now the automated urine sediment analysis UN2000 has made it possible to detect RTEC in large quantities with P/C and RTEC being two newly added parameters for urine detection by the machine. However, there are few literatures on the accuracy evaluation of UN2000 in detecting these two parameters though there have been many studies on that in detecting erythrocytes and leucocytes and the accuracy of urinary tract infections [15–18]. Therefore, in our study, 620 urine samples were tested to evaluate the accuracy of UN2000 in P/C and RTEC.

The result showed that the consistency of UN2000 in the P/C test and AU5800 was very high (Kappa = 0.858). In order to shorten the retention time of urine, preventing the destruction of visible components in urine to ensure the accuracy of RTEC detection results, urine samples retained within 2 h were selected in this study. On the accuracy evaluation of RTEC, the study established the reference range for healthy women (0-2.5cells/µl) after the detection of 336 healthy women. This range is larger than the reference range (0-1.7 cells/µl) established by Yuan Jinling et al[11] for the possible reason that only urine samples from healthy women were selected in this study while those from healthy men and women were selected by Yuan Jinling et al. The physiological structures of the urinary system of men and women are different, and there are differences in the reference range of urinary tangible components such as RBC, WBC, and EC whereas the reference range of men is mostly narrower than that of women[19, 20]. Therefore, the reference range in our study was relatively large, and there was a high consistency of UN2000 and conventional microscopic examination in RTEC(Kappa = 0.673).

To sum up, the P/C of UN2000 can be used as an alternative to biochemical detection and although the accuracy of RTEC is slightly lower than that of P/C, it still accurately reflects the level of RTEC in urine.

Our study further evaluated the application of these two new detection parameters in screening LN by comparing RTEC levels between the three groups. In LN group, the level was found to be significantly higher than that of the SLE group and of the control group(*P*<0.0001). There was no significant difference in RTEC level between the control and SLE groups(P = 0.094). The best cut-off value of RTEC screening LN of 1.35 cells/µl, with the AUC being 0.777 (95%CI = 0.702–0.853), is lower than the 2.5 cells/µl established in the study in which the value, therefore, is not applicable in the disease group although the Youden index is the highest. Thus, the study further screened cut-off values higher than 2.5 cells/µl and calculated the corresponding diagnostic efficiency. Table 3 shows that when the cut-off value is 2.8 cells/µl, the Youden index, negative predictive value and compliance of screening LN are the highest. Consequently, 2.8 cells/µl is more suitable than 1.35 cells/µl for screening LN from SLE patients.

Table 4 shows that the specificity, positive predictive value and coincidence of screening LN by P/C alone are the highest (97.50%, 96.46%, and 93.31%, respectively), while the sensitivity and negative predictive value are the highest when either P/C or RTEC is positive (95.97% and 96.24%, respectively). The results show that the ability of screening LN by P/C alone is better than that by RTEC alone, which may be related to the diagnostic criteria of SLE and P/C. Significantly, when either RTEC or P/C is positive, the sensitivity and PPV of screening LN are higher than those of single P/C screening (sensitivity increased from 87.90 to 95.97% and NPV rose from 91.23 to 96.24%). Therefore, the study believes that screening united with RTEC can more effectively exclude LN. In clinical practice, clinicians, depending on their own purpose, can choose the appropriate screening protocol for screening samples suspected of LN or for excluding LN. In addition, the same group of patients was selected when establishing and verifying the screening protocol in this study, which has limited the research conclusion. Next, the study will expand the number of urine samples to better verify this screening protocol.

## Data Availability

All data generated or analysed during this study are included in this published article.

## Abbreviations

P/C: protein/creatinine ratio.
RTEC: renal tubular epithelial cells.
LN: lupus nephritis.
SLE: systemic lupuserythematosus.
ESRD: end-stage renal disease.
S-M: sternheirnermalbin.
AUC: area under the curve.
NPV: negative predictive value.
PPV: positive predictive value.
SLICC: systemic lupus erythematosus international lupus research clinical collaboration group.
